# Plasmonically enhanced Fe(ii) coordination complexes allow SERS readout of spin state switching below the optical diffraction limit

**DOI:** 10.1039/d5sc06811h

**Published:** 2026-01-14

**Authors:** Yingrui Zhang, Zoi G. Lada, Wafaa Aljuhani, Yijun Lu, Chunchun Li, Yikai Xu, Grace G. Morgan, Steven E. J. Bell

**Affiliations:** a School of Chemistry and Chemical Engineering, Queen's University Belfast, University Road Belfast BT7 1NN UK s.bell@qub.ac.uk; b Department of Chemistry, University of Patras Rio Patras 26504 Greece; c School of Materials Science and Engineering, East China University of Science and Technology Shanghai 200237 P. R. China; d Key Laboratory for Advanced Materials and Feringa Nobel Prize Scientist Joint Research Center, Frontiers Science Center for Materiobiology and Dynamic Chemistry, School of Chemistry and Molecular Engineering, East China University of Science and Technology Shanghai 200237 P. R. China; e School of Chemistry, University College Dublin Belfield Dublin 4 Ireland

## Abstract

Creating and monitoring spin crossover (SCO) materials at the nanoscale is challenging since the spin transition phenomena are perturbed and methods for monitoring them are limited. Optical approaches for monitoring nanoscale SCO are attractive but limited by weak signal levels. Here, we demonstrate for the first time that surface enhanced Raman spectroscopy (SERS) allows enhanced readout of spin state transitions of even <1 µm SCO nano-objects confined within plasmonic nanovoids. Pressing dry crystalline [Fe(Htrz)_2_(trz)](BF_4_) (1) into the nanogaps between sheets of metal nanoparticles gave strong SERS signals but was unsuccessful since the surface perturbed the spin transition behaviour. However, when 1 was placed in the plasmonic hotspots between the Au cores in clusters of Au@SCO core–shell nanoparticles, SCO was retained and could be monitored using SERS. Importantly, the clusters showed thermal hysteresis loops which, although narrower than that of bulk 1 (9 K *vs.* 40 K), demonstrated that cooperative behaviour was retained in the nanovoids.

## Abstract 2

SERS has been used to observe temperature-driven spin crossover of an Fe^II^ coordination polymer confined in the nanovoids of <1 µm clusters of Au@SCO core–shell nanoparticles. Importantly, hysteresis is retained within the ∼nm hotspots.

## Graphical abstract

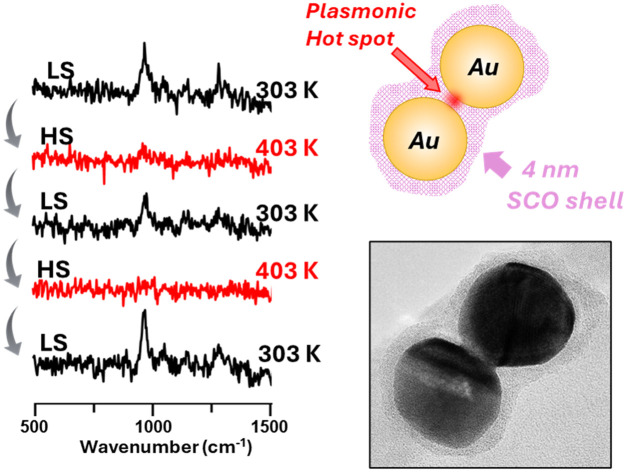

## Introduction

SCO materials have long been promoted due to their huge potential as components within the next generation of optoelectronic^1^ and memory devices^2–4^ but there has been no significant breakthrough to carry the phenomenon to the level of a device. In the last 15 years, this has led to intensive effects to prepare SCO nanoobjects and to address them thermally, magnetically or optically.^5–8^ However, reducing the size of the SCO materials may lead to loss of their key properties. For example, it has been found that preparing SCO materials as thin layers, which are nanoscale in 1 dimension, can cause them to be locked in a single spin state due to interactions with the surface.^9^ Even if SCO is retained, hysteresis effects, where the transition temperatures for switching LS-HS and HS-LS are different, may be reduced or lost at the nanoscale. This is also important because hysteresis is essential for SCO-based memory devices.^2–4^ Since hysteresis is widely believed to be associated with cooperative elastic lattice interactions, which arise in bulk crystalline SCO materials,^10^ it would also be expected to be affected as the size is reduced. Indeed, there are numerous examples where the hysteresis loops of SCO nanoparticles are narrowed compared to the bulk materials, although the fact that the effects have complex origins means that it is extremely difficult to predict the extent of the effect.^11,12^ Indeed, in some cases, the hysteresis has been found to increase for nanoparticles in a rigid host.^13^ Moreover, it has recently been shown that the memory effect could be observed even at the single molecule level where cooperative effects are not possible. In that very specific case the loss of lattice elastic coupling was replaced by intramolecular interactions within a constrained molecular structure.^14^ These general observations suggest that creating nanoobjects, which retain the desirable properties of the bulk materials, is not simply a matter of developing processes that allow the size of the materials to be reduced to the required level.

The second major challenge in studying SCO in nanoscale objects is to find a way to monitor the spin-state transitions, since reading out the signals from individual nanoscale samples requires the development of probe methods with extremely high sensitivity. Of course, if the samples are nanoparticles for example, it is straightforward to simply use conventional bulk techniques, but this only gives ensemble averages rather than addressing individual particles, a prerequisite for many applications. There are numerous potential approaches that might be used to monitor nanoscale SCO, since spin-state transitions are accompanied by significant changes in physical properties. Most obviously, these include changes in magnetic moments,^15^ but spin transitions also result in large changes in molecular volume,^6,16^ optical absorption,^7,17,18^ mechanical properties^19,20^ and vibrational modes.^17^ Among these options, optical probing is attractive because it enables non-invasive and rapid monitoring of molecular structure and electronic state changes. Similarly, IR and Raman techniques,^21,22^ which are also cost-effective and operationally simple, can be used to detect spin-state transitions *via* changes in vibrational fingerprints. Unfortunately, all these methods suffer from the fact that reducing the volume of the sample dramatically reduces the signal size, rendering these otherwise convenient techniques impractical for nanoscale systems. It has been shown that the high signals available from fluorescent molecules can allow detection of SCO in individual ∼200 nm nano-objects by doping the SCO material with fluorescent dye. Unfortunately, since this method relies on indirect sensing of the transition, it lacks the molecular specificity of methods based on electronic or vibrational spectroscopy.^23^ This is also true for surface plasmon resonance microscopy (SPRM), where changes in the optical contrast of SCO in individual particles can be used to detect SCO for particles in the size range of 100s of nm, which has been extremely successful in moving beyond ensemble properties to measurements of single particles.^7,18^ Finally, techniques specifically designed for probing samples at the nanoscale, such as scanning tunneling microscopy (STM)^9^ or four-dimensional electron microscopy,^24^ may be used for monitoring SCO, but these place stringent requirements on the sample and require complex instrumentation.

An attractive approach, which can retain the speed and simplicity of optical probes with high sensitivity and molecular specificity, is to couple the changes in vibrational spectra that accompany spin state transitions with the huge enhancement of electric fields proved by plasmonic nanogaps.^25–27^ This could potentially give a method for reading out spin crossover with such high sensitivity that it could be used to detect changes in spin within nanoscale volumes *i.e.* SCO-SERS. Plasmonic adsorption has been used to induce photothermal switching in Au@SCO nanostars,^28^ while previous attempts to use plasmonic effects to detect SCO with SERS have either used SCO films deposited on SERS-active substrates or assemblies of gold arrays linked by bridging SCO molecules to detect the spin crossover transitions.^29,30^ However, in the pioneering work on deposited films, it was not possible to detect spin state changes, since there was no clear change in their SERS spectra with temperature.^29^ An elegant study, which did allow spin state changes to be detected by SERS, used individual metal complexes to bridge the enhancing particles but of course this eliminated the possibility of creating cooperative SCO effects.^30^

Here, we have used [Fe(Htrz)_2_(trz)](BF_4_), 1, as the spin switching material, which was confined in the nanogaps between Ag or Au nanoparticles that give the high plasmonic fields needed for enhancement. The first approach to prepare the nanoconfined SCO material by directly pressing solid 1 into the plasmonic hot spots within arrays of Ag nanoparticles gave samples that showed strong SERS signals. However, the spectra of the pressed material did not show any evidence of thermally-induced spin state switching over a broad temperature range. We have found evidence that this is due to strong interactions between 1 and the Ag surface. To reduce the interference from the Ag surface, the plasmonic cavities were prepared by growing shells of the SCO material on Au. Preparation of clusters of Au@SCO core–shell nanoparticles with shells of 1 on the surface of Au nanoparticles gave samples in which the plasmonically active interparticle regions were filled with complex 1. These materials gave SERS signals that allowed the desired spin state switching in the expected temperature region to be followed. The SCO transition showed a hysteresis loop that was narrower than that of the bulk material, but was still readily detectable, which is striking, considering that the size of the interparticle gap (<2 nm) corresponds to the distance separating just 5 Fe(ii) centers in the crystal.^31^ The SCO-SERS signals were sufficiently intense to allow optical detection of spin state switching of particles with diameters below the optical diffraction limit. This work demonstrates not only that SCO-SERS is possible, but also that it can provide a powerful nanoscale probe of SCO phenomena near ambient temperature and on length scales that are appropriate for devices utilizing spin state transitions.

## Results and discussion

### Characterization of bulk SCO compound [Fe(Htrz)_2_(trz)](BF_4_)

The extensively researched iron(ii) coordination polymer, [Fe(Htrz)_2_(trz)](BF_4_), 1, was chosen for several reasons: (i) the compound exhibits large and abrupt thermal hysteresis occurring near room temperature, (ii) it demonstrates high thermal and environmental stability, allowing it to undergo multiple cycles of spin transition without significant degradation, and (iii) it is easy to synthesize and its properties can be easily tuned.^2,11,31,32^

**Fig. 1:**
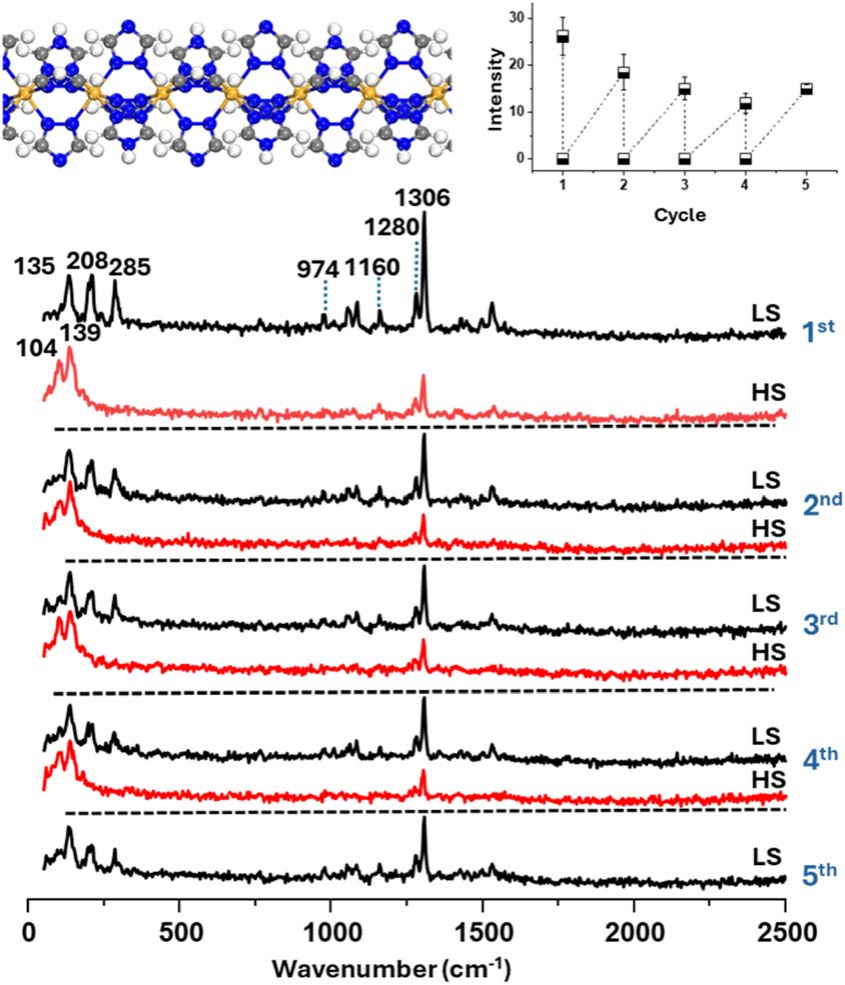
Characterization of thermally induced spin transition behaviour of bulk [Fe (Htrz)_2_(trz)](BF_4_) by Raman spectroscopy. Raman spectra of bulk [Fe(Htrz)_2_(trz)](BF_4_) collected over four heating–cooling cycles (303–383–303 K), showing reproducible spin-state switching behaviour. The intensities of the spectra are unscaled. Insets show the molecular structure of the SCO compound, which is a 1-D polymer (left), and variations in peak intensity at 285 cm^−1^ over four thermal cycles covering the spin transition temperature range (right).

Fig. 1 and S1 show background information on 1, which is useful in this context. Fig. 1 shows the structure of [Fe(Htrz)_2_(trz)](BF_4_), which is composed of polymer chains that crystallize as micron dimension rods. The sample used in this study was established by XRD (Fig. S1A and B) to be polymorph I.^32^ Magnetic susceptibility measurements (Fig. S1C) show a hysteresis of 40 K, similar to the results of differential scanning calorimetry (DSC) (Fig. S1D). Consistent with the literature,^31^ variable temperature Raman measurements of bulk 1 show large changes in the low frequency region associated with conversion of low-to high-spin (LS-HS) forms. Here, we show data, which demonstrate that the changes are reproduced over 4 temperature cycles, with notable variations in the intensity of the peak around 285 cm^−1^, as shown in the inset of Fig. 1.

### Pressing the bulk SCO compound [Fe(Htrz)_2_(trz)](BF_4_) into Ag nanoparticle arrays

**Fig. 2:**
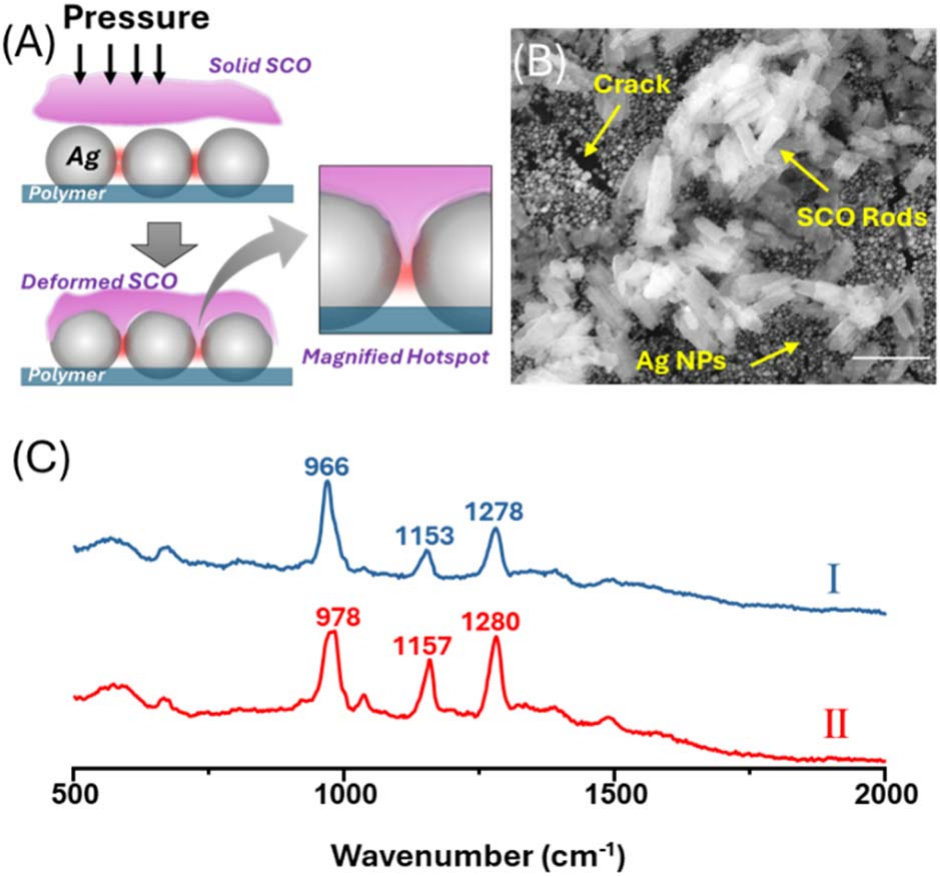
Pressing spin-crossover materials into plasmonic nanocavities enables localized SCO-SERS readout. (A) Schematic illustration of the process of pressing solid SCO materials into the plasmonic nanocavities between Ag nanoparticles of the enhancing substrate. (B) SEM image of a sample prepared by pressing crystals of 1 into a substrate composed of a sheet of Ag nanoparticles fixed to a polymer support. The image shows that the rods of 1 have fractured during the mechanical pressing process. The scale bar is 1 µm. (C) Examples of the two distinct types of SERS spectra obtained at different points on the pressed SCO sample.

The challenge for obtaining SERS spectra of 1 is that, since it is a polymer chain, it cannot simply be dissolved into solution and mixed with the enhancing material because this might lead to free triazole ligand, rather than crystalline 1, being adsorbed onto the surface. Triazole ligands are known to have strong affinity for metal surfaces.^33^ For this reason, here we pressed dry solid crystalline 1 into a SERS substrate composed of a planar array of closely packed Ag nanoparticles, which show a broad plasmonic extinction at 785 nm (Fig. S2), as illustrated in Fig. 2A. The objective was to directly place the crystalline 1 into the enhancing plasmonic hot spots without using solvent. In this case it was important that the SERS enhancement of the crystal sample was so large that the observed spectrum would be dominated by the nano volume of material located within the enhancing hot spots, rather than the remaining bulk solid sitting on top of the substrate. Fig. 2C shows spectra obtained for pressed SCO samples, the signals are clearly SERS because the spectra are very different from those of bulk 1 in either high- or low-spin states, which are shown in Fig. 1. The two most prominent bands have previously been assigned to in-plane *δ*(C–H) ring deformation (*ca.* 977 cm^−1^) and in-plane *ν*(C–N) and *ν*(N–N) stretching (*ca*. 1280–1307 cm^−1^).^31^ Point sampling at random locations over the sample showed that signals were not confined to isolated regions of the surface but could be obtained at any position where the crystals had been pressed into the substrate. Two slightly different types of spectra were observed at different points on the surface, one showed three distinct bands at *ca.* 968 ± 3 cm^−1^, 1155 ± 2 cm^−1^ and 1280 ± 2 cm^−1^ (Type I spectra), and the second showed bands at 979 ± 1 cm^−1^, 1156 ± 1 cm^−1^ and 1281 ± 2 cm^−1^ (Type II spectra). This obvious heterogeneity may be due to the fact that the sample is pushed into hot spots, which could cause parts of the crystal structure to be degraded. Indeed, the SEM image (Fig. 2B) of the sample shows clear evidence that the crystals on the surface have fractured under pressure, although we cannot image the crystals within the nanovoids directly. The Type I spectrum is very similar to those obtained when free ligand is dropped from solution onto the surface or more importantly, when crystalline ligand is pressed onto the surface (see Fig. S3). This suggests that Type I spectra observed for pressed 1 are due to 1,2,4-triazole ligand breaking away from the polymer chain and adsorbing to the Ag surface in the hotspot. This is reminiscent of previous studies where it was found that spin-crossover complexes (Fe(neoim)_2_) can remain intact on certain metal surfaces such as Ag (111) but may undergo fragmentation on Au (111), due to stronger metal–ligand interactions and substrate-induced distortions.^34^ If the Type I spectra are due to the ligand fragment, they would not be expected to show a spin transition, and variable temperature studies carried out over the appropriate temperature range (302–382K) indeed show no change in the observed signal (Fig. S4A and C).

Variable temperature SERS experiments were also carried out on the Type II species observed in the pressed samples (Fig. S4B and D), since it was possible that this was intact crystalline 1 present in the hotspots and could display thermal SCO behaviour. However, as Fig. S4B and D show, no changes in the positions or relative intensities of the bands were observed over several heating/cooling cycles covering the SCO range. This could be due to spin locking,^35^ which is widely observed for thin layers of SCO compounds on metal surfaces and is typically attributed to interactions with the substrates perturbing the properties of the complex or even causing it to fragment. Fragmentation was discussed above and is treated in more detail in Section 2 of the SI. In the current study, it is possible that when 1 is pressed against the Ag surface the ligand detaches from the crystalline material (and may be detected as discussed above) while the residual complex has a different chemical composition and does not display the SCO properties of the parent complex.

**Fig. 3:**
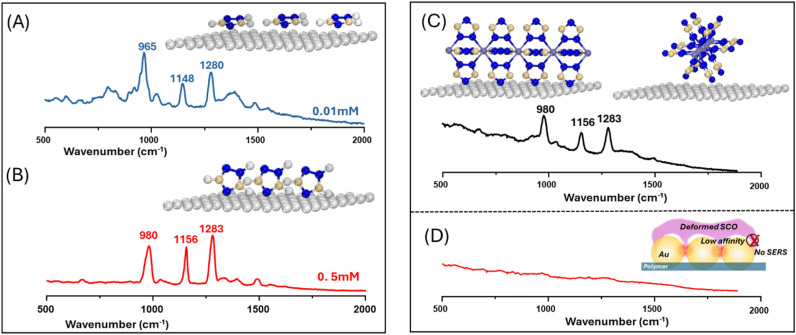
Ligand orientation at the metal interface governs the SERS response in solution and in the solid state. (A and B) SERS spectra of 0.01 mM and 0.5 mM triazole ligand enhanced by aqueous Ag colloids. The inserts illustrate structures where the ligands lie flat on the Ag surface at low concentration (0.01 mM) but sit at a more upright orientation at higher concentration (0.5 mM). (C) SERS spectra of dry 1 pressed onto an Ag film. The inset illustrates how the structure of the 1-D polymer means that, when it sits on the Ag surface, the ligands near the surface have an upright orientation. (D) The Raman signal obtained by pressing 1 into an Au film, which shows there is negligible SERS enhancement. The inset is a cartoon illustrating the weak interaction between solid 1 and the Au nanoparticle surface.

An alternative explanation is that when compound contacts the Ag atoms of the surface, it remains intact, but its properties are perturbed by strong interactions with the metal substrate. This has been observed for layers of [Fe(3,5-(CH_3_)_2_Pz)_3_BH)_2_] on Cu(111), where complex changes in the hysteresis behaviour were observed for samples that were several layers thick. This was attributed to the balance between interfacial constraints and cooperativity between the layers.^36^ In the case of 1 on Ag, it is striking that the structure of the polymer means that the nitrogen heteroatoms at the 4 position point away the Fe atoms running along the central axis and are oriented so that they can coordinate to the Ag ions on the surface, as illustrated in Fig. 3C. If this geometry was adopted, it would be expected to strongly perturb the electronic structure of the complex and could lead to spin locking. Supporting evidence for this model comes from SERS studies of the free ligand recorded with simple aggregated Ag colloids, where the signals taken at low concentrations, which are characteristic of a flat orientation (Fig. 3A), are similar to Type 1 spectra recorded for pressed 1. Similarly, those at high concentrations, which are more upright (Fig. 3B), resemble Type II. This is discussed in more detail in Section 2 of the SI.

Irrespective of the detailed mechanism, the clear observation is that pressing 1 into nanovoids in Ag nanoparticle arrays gave samples that showed strong SERS signals, but the strong interactions with the Ag surface perturbed the electronic structure to the extent that the SCO behaviour characteristic of the bulk compound was not observed over the temperature range investigated *i.e.* no SCO-SERS was detected. This could be a general problem in SCO-SERS, since SERS is intrinsically a surface (or at least, near surface) effect, while previous studies on SCO using other approaches have shown that the surface can dramatically affect the SCO properties.^37–39^ This means that the possibility of observing the potentially useful bulk-like properties in nanoscale SCO materials within plasmonic cavities using SERS could potentially be prevented either by general size effects (where reducing SCO materials to nm dimensions causes loss of cooperativity) or by SERS-specific perturbations associated with placing SCO at the enhancing metal surfaces.

In an attempt to reduce the metal–complex interactions at the surface, similar experiments were carried out on Au nanoparticle arrays prepared in the same way as the Ag substrates used above. The data in Fig. 3D show a representative spectrum recorded for a sample where 1 was pressed onto a Au nanoparticle array. This spectrum is typical of those recorded over the entire contact region, since none of the spectra showed any detectable SERS signals from the complex, which indicates that complex 1 has low affinity toward Au and does not adsorb effectively. As demonstrated by Xu *et al.*,^40^ molecules with strong affinity toward Au or Ag nanoparticles produced immediate SERS signals upon direct pressing into plasmonic substrates. The lack of such a response in the Au case implies that complex 1 interacts more weakly with Au than with Ag.

### Au@[Fe(Htrz)_2_(trz)](BF_4_) SCO core- shell nanoparticles

**Fig. 4:**
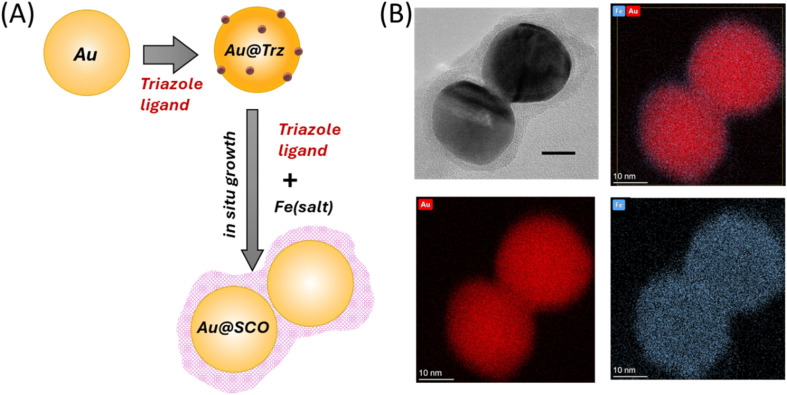
Preparation and structural characterization of Au@SCO core–shell nanoparticles. (A) Schematic illustration of the preparation of Au@[Fe(Htrz)_2_(trz)](BF_4_) SCO core–shell nanoparticles. In the first step, the Au nanoparticles are partially covered with triazole ligands, which bridge to the SCO shell created in the 2nd step. (B) TEM/EDX images of a core–shell dimer showing the distribution of Fe and Au, along with an overlayed image showing both elements.

**Fig. 5:**
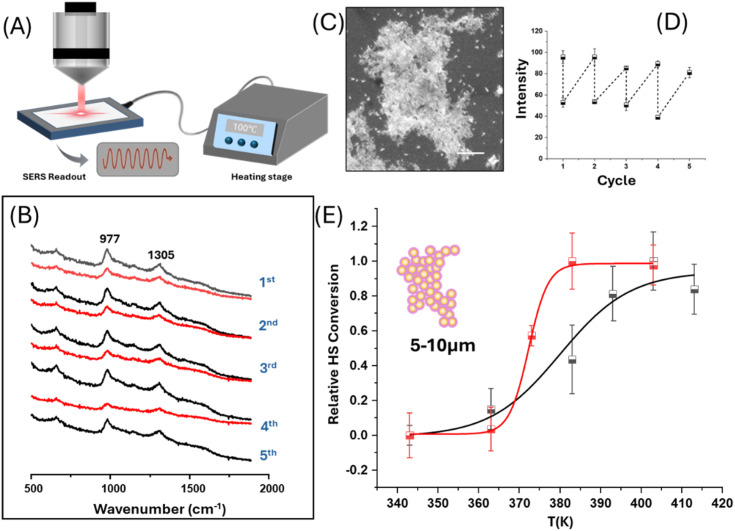
*In situ* SERS monitoring of thermally-induced spin transitions and hysteresis behaviour in large Au@SCO clusters. (A) Schematic illustration of monitoring the thermal-induced spin transition of large Au@SCO clusters with SERS. (B) SERS spectra of spin transition behaviour of the large *ca.* 5–10 µm Au@SCO cluster (SEM image shown in (C)), recorded during 4 heating and cooling cycles (303–413–303 K). (C) SEM image of the Au@SCO cluster used in the thermal cycling experiments. The scale bar is 1 µm. (D) Plot showing variations in peak intensity at 977 cm^−1^ during the thermal cycling process shown in (B). (E) Curves showing the hysteresis loop in the thermal spin transition by plotting the relative HS conversion calculated from SERS intensity at several temperatures during heating (black) and cooling (red) processes. Each data point represents the average of five repeated measurements recorded while cycling between the initial and target temperatures (see text). Error bars are ±1*σ*.

To overcome the limitations created by the weak interactions between Au and 1 for SERS measurements, the SCO complex was grown as a thin shell around Au nanoparticles to ensure that the complex was in close proximity to the enhancing surface. The Au@SCO core–shell nanoparticles were synthesized following a method adapted from Torres-Cavanillas *et al.* with minor modifications;^2^ in particular, the size of the Au nanoparticles was changed from 12 nm to 28 nm to improve the plasmonic properties of the product (Fig. 4A). First, citrate-stabilized Au nanoparticles were treated with 0.5 mM 1,2,4-triazole ligands, which led to partial ligand exchange, as evidenced by a decrease in zeta potential from −35.7 ± 1.0 to −30.9 ± 0.6 mV and by the SERS intensity data (see Fig. S5). This partial ligand exchange provided the nucleation sites, which are essential for the subsequent growth of the SCO shell.^2^ TEM/EDX images in Fig. 4B and S6 confirm the formation of well-defined core–shell structures, where the SCO shell has an average thickness of 3.6 ± 1.1 nm. These core–shell particles form as clusters with an average diameter of 105 nm, as shown by dynamic light scattering measurements (Fig. S7). This is a much larger diameter than can be accounted for by the size of the original particles (28 nm) plus the shell thickness (2 × 3.6 nm). The TEM images show that the separation between the particles in the aggregates is much less than the combined thickness of their shells (Fig. S6). This is particularly clear in Fig. 4B, which shows a simple dimer where the separation is less than the thickness of even a single shell. This suggests that aggregation occurs during shell formation rather than after the shells have completely formed around all of the particles, which is consistent with the zeta potential of the particles falling as the particle shell grows (initial value −30.9 ± 0.6 mV and final value −4.7 ± 0.3 mV). The UV-Vis extinction spectra (Fig. S8) are dominated by the surface plasmons of the particles and show significant broadening and a large red shift, which is consistent with strong coupling between the Au particles in the aggregated core–shell clusters. This coupling creates the localized areas of high electric field enhancement (hot spots), which are necessary to give SERS signals of the SCO compound sitting at the junctions between the particles. Fig. 5B shows SERS spectra of a “bulk” sample of core–shell particles, obtained by focusing a near diffraction limited (∼1 µm radius, Section 3 in SI) laser spot onto a large (*ca.* 10 µm) aggregate (Fig. S9) created by drying a suspension onto an aluminum foil support. The spectra are weaker than those obtained with the pressed samples on the particle arrays discussed above, but they are similar to them since they also show two distinct bands in approximately the same positions (see Fig. 2C and 5B). However, the bands in the Au@SCO core–shell samples are at *ca.* 977 cm^−1^ and 1305 cm^−1^, so the higher wavenumber band is significantly shifted from its position on the particle arrays (*ca.* 1280 cm^−1^), although it is in a similar position to the strongest band of the normal Raman spectrum of 1, which is at 1306 cm^−1^. These observations suggest that the spectra of Au@SCO core–shell samples are different from the pressed samples because the *in situ* growth process creates intact shells of 1.

### Characterisation of SCO-SERS in large clusters

The SCO behaviour of the Au@SCO nanoparticles was investigated using variable temperature SERS experiments, as illustrated in Fig. 5A. Fig. 5B shows data for a large aggregate (Fig. 5C) taken well below and above the expected transition temperature. The spectra show there is a large overall intensity decrease at high temperature, although there is no significant change in the band positions. The process was reversible, as it was observed in spectra recorded over several cycles. This effect was not observed in control experiments where only the ligand was included in the shell (see Fig. S10). It has been known since the early Raman studies by Bousseksou and co-workers that LS-HS switching produces characteristic changes in both vibrational frequency and intensity.^41^ More specifically, for the [Fe(Htrz)_2_(trz)](BF_4_) system, Urakawa *et al.*^31^ showed that LS-HS SCO leads to the disappearance of several ligand-based modes in the 700–1400 cm^−1^ fingerprint region and to pronounced softening of Fe–N vibrations below 400 cm^−1^, arising from ligand-field change and elongation of the Fe–N bond in the HS state. We also found similar changes in the bulk spectra, as shown in Fig. 1 above. Consistent with these signatures, the characteristic triazole ring vibrations near 977 and 1305 cm^−1^ in our SERS spectra show a clear and reversible intensity decrease upon heating and cooling. Unfortunately, the low wavenumber region is masked by a strong background signal in the SERS experiment, which prevents observation of the low frequency bands, which are characteristic of SCO in bulk 1. However, the overall decrease in intensity in the higher wavenumber region observed in the SERS data, which is also shown for LS-HS conversion in bulk 1, is sufficient to clearly indicate that LS-HS conversion is being observed.

Measuring the transition temperature by directly recording SERS spectra at a series of increasing and decreasing temperatures was difficult due to drift in the overall signal intensity with time. This problem was addressed by making a series of repeat measurements while cycling between the initial and target temperature (as shown in Fig. 5B) and measuring the height of the largest band at 977 cm^−1^. A plot of these data (Fig. 5D) shows that the change in signal induced by stepping the temperature up or down can be measured, even if the overall signal drifts during the measurements. Repeating this process for a range of target temperatures and carrying out the process with either 303 K or 413 K as the initial temperature (Fig. S11 and S12) allowed a complete heating and cooling curve to be plotted, as shown in Fig. 5E. This plot clearly shows the spin interconversion occurs around 375 K with a hysteresis of *ca.* 10 K. In this plot, the *Y* axis is the relative HS conversion, which is the proportion of the total observed change found at a given temperature. The spin interconversion parameters can be determined more accurately by fitting the data using a Boltzmann function, as described in Table S1, which produces the fitted lines shown in Fig. 5E and gives transition temperatures of 381 K for heating and 372 K for cooling. These are similar to the bulk values of 381 K and 342 K, but the thermal hysteresis width of 9 K in the nanosized SCO thin shell is significantly narrower than the 40 K observed in bulk materials.

It is important to note that in these experiments, although the volume of the sample, which is monitored, is determined by the laser spot size, the probed volume contains numerous hot spots, so the measured SCO behaviour represents an ensemble average of all these nanovolumes. The size of the plasmonically-enhanced region between the particles is determined by the thickness of the shell layer that sits between the particles. The upper limit for this distance would be for two particles with complete shells in contact, which is 2 × 3.6 = 7.2 nm, but the TEM images clearly show that the distance between the particles is typically <2 nm, which is consistent with the requirement for SERS enhancement that the target molecules are within a few nm of the surface. The probed volume is significantly smaller than in previous experiments on crystalline SCO materials, and although it is obviously larger than that of single molecule experiments,^30^ these are not possible for polymeric compounds such as 1. The observation of a hysteresis loop, which is narrower than that of the bulk material, is consistent with other studies of SCO, which probed the averaged ensemble properties of a large number of very small dimension individual objects. For example, Ramón *et al.* used DSC to probe the properties of bulk samples of core–shell particles of 1 with Au cores and found that the SCO hysteresis loop was 19 K, demonstrating that reduction of the SCO layer to a thickness of *ca.* 4 nm did not completely eliminate hysteresis.^2^ Similarly, Monica *et al.* studied pure nanocrystals of 1 at various sizes and found that the width of the hysteresis loop decreased from 38 K to 24 K as the particle size decreased from 16 nm to 4 nm.^11^ These changes in SCO properties with size can be associated with simple physical effects, since in smaller nanoparticles a larger proportion of the Fe centers lie at the surface, where they have fewer neighbors and experience weaker intermolecular interactions. As a result, the spin transition can appear more gradual, reflecting the reduced cooperativity that is intrinsic to size reduction. However, it is well known that attributing changes in SCO behaviour to pure size effects is difficult because size reduction may be accompanied by changes in the morphology, crystallinity, and composition of the samples.^6^ In addition, sample matrix effects can be important. For example, [Fe(Htrz)_2_(trz)](BF_4_) nanoparticles embedded in a silica monolith showed an unusually wide 65 K hysteresis, which was attributed to elastic coupling with the rigid host matrix.^13^ Similarly, in core–shell particles, coupling between the rigid core and SCO interface can give perturbations associated with interface stress, which is effectively a special case of the general matrix effects.^42,43^ For the current work it is possible to judge the importance of matrix effects in the SCO particles by comparing the results of previous studies on 4 nm particles of pure 1 and Au@SCO core–shell particles of 1 with 4 nm shell thickness. In both these cases, the changes in thermal properties from the bulk were extremely similar (4 nm particle *T*_1/2_ = 342/367 K, core–shell *T*_1/2_ = 342/361 K),^2,11^ suggesting that the effect of the Au core on the SCO properties of Au@SCO core–shell particles of 1 is small. This is consistent with the results of the experiments pressing 1 into Au films discussed above. If the matrix and interface effects are negligible in our samples, then the observed narrowing of the hysteresis loop in our Au@SCO core–shell system can primarily be attributed to pure size reduction effects and the increasing importance of surface phenomena at the dimensions being probed. It is not surprising that pure size effects might be observed in our systems, since the Fe⋯Fe distance in the [Fe(Htrz)_2_(trz)](BF_4_) polymer chain is approximately 0.4 nm,^31^ meaning that the length scale of the SERS-detectable region, which is typically below 2 nm, corresponds to the distance separating just five Fe^II^ centers in the crystal. Therefore, a significant proportion of the centres in the probed volume are at or near the surface. This means that it is not hysteresis narrowing, but the fact that hysteresis is detected at all, which is the most striking observation, given the low numbers of centres involved in the potential cooperative behaviour.

### Observation of SCO-SERS in <1 µm clusters

Ultimately, the driving force for many recent SCO studies has been to observe and exploit SCO phenomena at increasingly small length scales. It is possible to characterise the effect of reducing size by creating large numbers of small objects (*e.g.* individual nanoparticles or particles confined with a host matrix). The ensemble properties of these bulk samples may then be monitored using any of the available approaches with the appropriate sensitivity. In the case of particles in host matrices or core–shell particles, the proportion of the bulk sample, which is composed of the SCO material, may be relatively small and this may place demands on the sensitivity required, but the total amount of sample can be increased to compensate. However, this approach is very different from switching and probing individual objects on the nanoscale, which may be required for applications of the SCO materials and also allows the averaging effects of ensemble measurements to be reduced.^7,18^

The core problem is that the sensitivity of many measurement approaches is too low to probe individual nanoscale objects. This means that experiments demonstrating switching in <1 µm objects are rare, although at the smallest length scales tunnelling measurements can be carried out on individual molecules. The challenge is in carrying out measurements in the size range where cooperative effects may, or may not, occur, although notable recent studies based on monitoring changes in refractive index were able to demonstrate detection of SCO in <1 µm particles.^7,18^

**Fig. 6:**
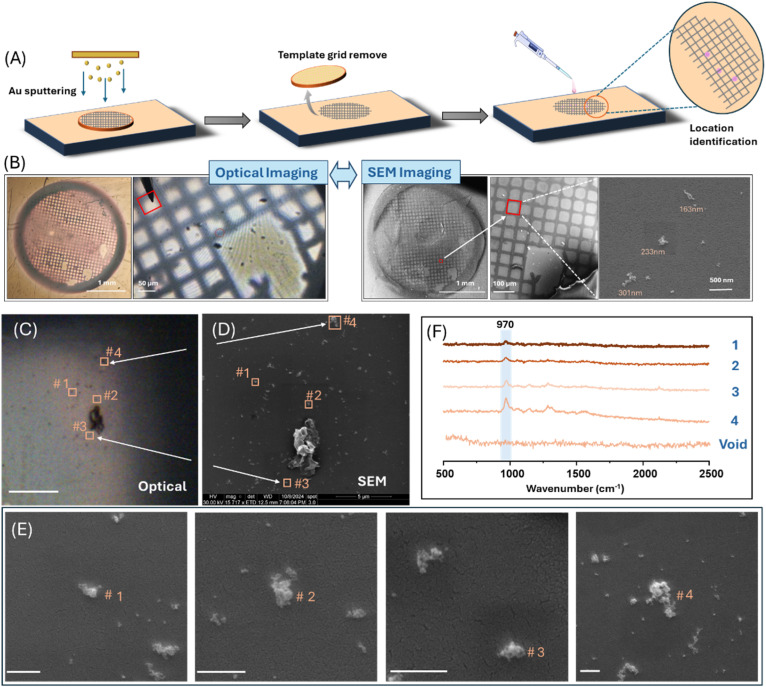
Correlated optical and SEM imaging enables tracking and spectral readout of individual Au@SCO clusters on patterned substrates. (A) Schematic illustration of the fabrication of a grid-patterned planar Au-coated glass support used for the identification and tracking of small Au@SCO clusters. (B) Comparison of the optical (left) and SEM (right) images of the complete template and higher magnification images of a section of the grid with deposited 1. (C–E) Illustration of visualization and tracking of small Au@SCO clusters using correlated optical and SEM imaging. (C) Optical image recorded with the SERS microscope showing multiple Au@SCO clusters. The positions of 4 points containing particles that are only just visible but whose SERS spectra were recorded are marked. The scale bar is 10 µm. (E) SEM images of each of the 4 tracked particles obtained at high magnification. All scale bars are 500 nm. (F) SERS spectra obtained from the four marked Au@SCO clusters, along with a control spectrum showing the empty sample areas gave featureless spectra.

**Fig. 7:**
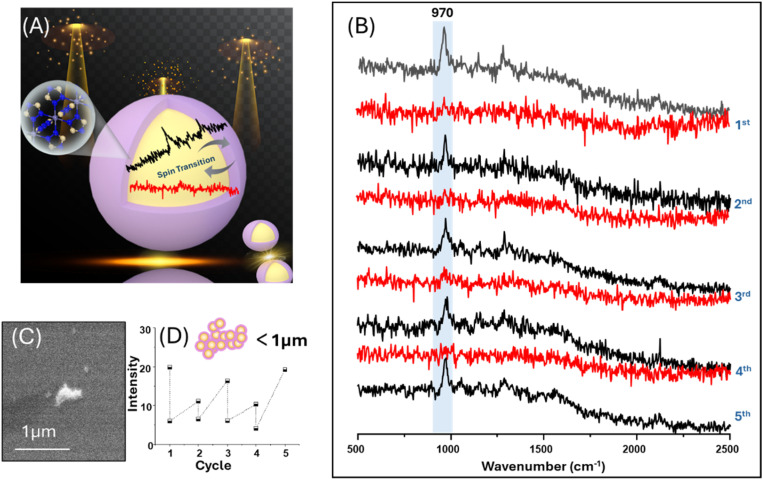
Reversible spin transition detected by SERS in a submicron Au@SCO cluster. (A) Schematic illustration of the thermally induced spin transition in a small Au@SCO cluster, featuring only a few Fe(ii) units in the plasmonically enhanced region of the shell, which lies between the particles. (B) SERS spectra of a <1 µm Au@SCO cluster recorded as the temperature was cycled between 303 and 403 K showing reproducible spin transition behaviour. (C) SEM image of the Au@SCO cluster whose SERS spectra are shown in (B). (D) Plot of the variations of the peak intensity at 970 cm^−1^ in the SERS spectra shown in (B).

In the current study, we have found that even with conventional Raman microscopy, the sensitivity of SCO-SERS is sufficiently high to allow detection of spin state transitions in small clusters, which are individual objects that have their largest dimension <1 µm and indeed can be sub-diffraction limited. As illustrated in Fig. 6A and B, in these experiments, a sample containing clusters with a broad distribution of sizes was deposited onto a gold-coated glass slide with a grid pattern to allow the location of particles to be indexed. Suitably sized (<1 µm) particles were chosen using optical microscopy within the Raman instrument and their SERS spectra were recorded before the samples were taken for higher resolution imaging by SEM. Fig. 6(C–E) shows the optical and SEM images of a sample where four distinct clusters were chosen. The particles were barely visible in the optical image due to their small size, but SEM imaging (Fig. 6E) showed they consisted of tens of individual nanoparticles with an island-like morphology. These clusters ranged in size from approximately 200 to 700 nm, but their high Raman scattering signals meant that SERS spectra could be obtained for particles, which were below the diffraction limit of the probe laser (Fig. 6F). Fig. 7 shows data for one of these small clusters, where four LS-HS cycles showed consistent loss and then recovery of the characteristic 970 cm^−1^ peak, indicating stable reversible SCO behaviour.

These experiments were on a similar length scale to those from Liu *et al.*^7,18^ who used SPRM to measure thermal hysteresis in single particles. In those experiments, it was also possible to detect SCO in particles as small as 200 nm; however, in that case, the whole object was composed of the pure compound while in the current experiments even the 200 nm cluster was composed of Au core–shell particles, so the volume of 1 that was probed is smaller than the overall cluster dimension would suggest. This is partly because only a fraction of the particle's volume is composed of the SCO shell, although in this case the particle geometry (28 nm core and 3.6 nm shell) means that approximately 50% of the particle's volume is composed of the SCO shell, so this is not a large effect. More importantly, in the SCO-SERS experiments, it is only the part of the sample that is in the plasmonic nanogaps is enhanced, and this is a significantly smaller fraction of the total. Section 4 in the SI shows the example of a particle that can be approximated as a densely packed two-layer Au@SCO cluster (*ca.* 278 × 189 × 70 nm). If it is assumed that all hot spots at the interparticle gaps within the cluster contribute to SERS enhancement, the upper limit of the probed volume can be estimated to be only ∼3150 nm^3^, corresponding to just 0.08% of the total cluster volume.

## Conclusions

The data shown here demonstrate that the very large signal enhancement given by SERS allows vibrational spectra of 1 to be recorded in clusters of Au@SCO core–shell particles, which are ensembles of numerous individual nanometric volumes of molecules confined within sub-2 nm plasmonic junctions, and that this is possible even for clusters with dimensions below the optical diffraction limit. Although SERS gives sufficient sensitivity to allow individual nanoscale objects to be probed, it does require close contact between the sample and the enhancing surface, which may perturb the spin transitions being measured. In the current study, pressing crystalline SCO materials into the plasmonic nanogaps between arrays of Ag particles generated strong SERS signals but variable temperature experiments failed to show any evidence of spin transitions. This was due to strong interactions between the complex and the Ag surface. Alternatively, confined SCO shell nanostructures, which were prepared on Au plasmonic cores, gave materials where there was close contact between the SCO material and the surface, but the interactions were expected to be weaker than with Ag. For these nanoclusters, variable temperature measurements showed changes in the SERS spectra associated with spin transitions (SCO-SERS) at similar temperatures to those observed for bulk 1, but with a narrower hysteresis loop due to reduced cooperativity in the nanomaterials. The effect is mainly attributed to pure size reduction effects, where a high proportion of Fe(ii) centres lie at or near the surface, weakening intermolecular interactions essential for maintaining hysteresis, although of course there may also be some coupling between the surface and the confined SCO crystal. In the current experiments, SCO transitions were probed for clusters ranging from 10 µm down to <1 µm. The smallest particles that could be detected were *ca.* 250 nm, which was set by the optical diffraction limit of the instrument and is similar to the smallest particles studied by SPRM methods.^7,18^ However, decreasing the size range of the SPRM measurements will be challenging due to the nature of the measurements, while we would expect that SERS measurements could be carried out on individual hot spots within confined SCO materials using tip-enhanced approaches, which would drive the length scale down to <10 nm. In general terms, the size range of plasmonic hot spots used in SCO-SERS is well-matched to studies that investigate the limits of cooperative behaviour, since the enhanced volume is so small that it can only contain relatively few metal centers. More broadly, since this work demonstrates that plasmonic enhancement can be used to read out spin state switching on the nanoscale, it opens numerous possibilities both for further fundamental studies and design of devices based on SCO-SERS.

## Author contributions

Conceptualization, S. B.; methodology, Y. Z., Z. L., W. A., Y. L., C. L., Y. X., G. M., and S. B.; formal analysis, Y. Z.; writing – original draft, Y. Z.; writing – review & editing, S. B.; supervision, S. B.

## Conflicts of interest

There are no conflicts to declare.

## Supplementary Material

SC-OLF-D5SC06811H-s001

## Data Availability

The data supporting this article have been included as part of the supplementary information (SI). Supplementary information: materials and methods; fragmentation and orientation on metal surfaces; estimation of the laser spot size and hot spot volume; experimental characterizations including SEM; molar magnetic susceptibility; XRD; DSC; UV; SERS; TEM; DLS; and calculation method. See DOI: https://doi.org/10.1039/d5sc06811h.
