# Supplementary material for: Plasmonically enhanced Fe(ii) coordination complexes allow SERS readout of spin state switching below the optical diffraction limit
Source: Chem Sci. 2026 Jan 14;17(9):4814–24. doi: 10.1039/d5sc06811h (PMC12810712; doi:10.1039/d5sc06811h)
Supplement: SC-017-D5SC06811H-s001 [file SC-017-D5SC06811H-s001.pdf]

Supplementary Information (SI) for Chemical Science.
This journal is © The Royal Society of Chemistry 2026

Plasmonically-enhanced Fe(II) Coordination Complexes Allow SERS Readout of Spin State Switching Below the Optical Diffraction Limit

*Yingrui Zhang[a], Zoi G. Lada[b,e], Wafaa Aljuhani[a], Yijun Lu[a], Chunchun Li[c], Yikai Xu[d], Grace G. Morgan[e] and Steven E.J. Bell[a]**

Section 1 Materials and Methods

Materials

Tetrachloroauric (III) acid trihydrate ($HAuCl_4 \cdot 3H_2O$) (99.9999%), silver nitrate ($AgNO_3$) (99.9999%), trisodium citrate dihydrate ($Na_3Ct \cdot 2H_2O$), tetrabutylammonium nitrate ($TBA^+NO_3^-$), ethanol, polystyrene, dichloromethane (DCM), iron tetrafluoroborate hexahydrate [$Fe(BF_4)_2 \cdot 6H_2O$], 1,2,4-triazole and 1,2,4-triazole sodium derivative were purchased from Sigma-Aldrich®. Distilled deionized (DDI) water with a low resistivity of 18.2 MΩ was used for all experiments.

Synthesis of bulk [Fe(Htrz)$_2$(trz)](BF$_4$) SCO material

The bulk [Fe(Htrz)$_2$(trz)](BF$_4$) compound was synthesized using the direct method with slight modifications[1]. Specifically, 1.7 mL of a 0.3 M [$Fe(BF_4)_2 \cdot 6H_2O$] solution was mixed with 1.7 mL of a 0.9 M triazole solution. Both solutions were prepared in a water/ethanol mixture with a volume ratio of 10:7. The resulting mixture was stirred at room temperature for 2 hours and then left to stand undisturbed for an additional 2 hours. Following this, the resulting mixture was then subjected to centrifugation at 5000 rpm for 5 minutes, which was subsequently washed three times: first with water, followed by two washes with a water/ethanol mixture. Finally, the [Fe(Htrz)$_2$(trz)](BF$_4$) was allowed to dry overnight in ambient conditions.

Synthesis of Ag (Au) surface-exposed nanoparticle sheets

This process can be found by our previous published work[2]. Briefly, a mixture of 5 mL citrate reduced-Ag (Au) colloid suspension[3, 4], 3 mL polystyrene/dichloromethane (0.6 g/mL) solution and 130 µL of aqueous $TBA^+NO_3^-$ (10^{-3} M) was shaken for 30 seconds. After that, the mixture was transferred to a polypropylene petri dish, and a lustrous metallic film subsequently formed at the liquid-liquid interface. Then, the film was left overnight at room temperature to allow all the DCM to evaporate, enabling a robust polymer-supported Ag (Au) nanosheet.

28 nm Au Colloid synthesis

The Au NPs with an average diameter of 28 nm were synthesized using the classic sodium citrate reduction method. Briefly, 4 mL of sodium citrate solution (0.5% w/v) was added into 250 mL of boiling $HAuCl_4$ solution (0.16 mM) while stirring vigorously. The solution was maintained at boiling temperature for 10 minutes, after which the heating was stopped, allowing the solution to cool naturally to room temperature. The resulting Au NPs solution was then stored in a refrigerator.

Synthesis of Au@ [Fe(Htrz)$_2$(trz)](BF$_4$) SCO core shell nanocomposites

Au@[Fe(Htrz)$_2$(trz)](BF$_4$) NP$_S$ were synthesized by Torre-Cevadilla's method with slight modifications[5]. Briefly, an aqueous solution of triazole (200μL, 0.5 mM) was first added to 5 mL of a AuNPs solution followed by vigorous stirring for 30 min to partial replace citrate on Au NPs surface with triazole ligand. Subsequently, aqueous solutions of the precursors of ([Fe (BF$_4$)$_2$·6H$_2$O]) (5 mL, 0.5 mM) and 1,2,4-triazole (5 mL, 1.5 mM), were simultaneously added at a continuous rate (0.08 mL/min) under stirring at room temperature. After that, the obtained solution was sonicated for 60s and allowed to stand until the solid settled to the bottom of the flask (typically overnight) when the supernatant was carefully removed. Fresh deionized distilled water (DDI) was added, and the mixture was sonicated again for 60 s before further use.

SERS characterization

SERS detection was carried out utilizing a WITec Alpha 300 R Confocal Raman Microscope equipped with a 785 nm laser. For experiments on characterization of spin transition behaviour of bulk [Fe(Htrz)$_2$(trz)](BF$_4$) SCO compounds in Figure 1 and experiments on illustrating the similarity of the SERS spectra of triazole ligand obtained from Ag films under 3 different experimental conditions in Figure S3, a 10× lens and 10 mW laser power, with an accumulation time of 30 seconds was used. For experiments on Ag colloid enhanced SERS spectra of 0.01/0.5 mM triazole ligand in Figure 3A and B, a 10× lens and 30 mW laser power, with an accumulation time of 30 seconds was used. For experiments on small SCO clusters <1 μm in Figure 6 and 7, a 100× lens and 0.3 mW laser power, with an accumulation time of 30 seconds was used. For experiments on step 1 of the preparation of core-shell SCO particles using the specified triazole concentration in Figure S5, a 10× lens and 60 mW laser power, with an accumulation time of 60 seconds was used. Other measurements were recorded using a Raman microscope constructed in-house based on a PerkinElmer microscope and a 785 nm diode laser (10× lens and 80 mW laser power, with an accumulation time of 30 seconds). Temperature-dependent Raman spectra were collected to study the SCO behaviour of the samples, using a Linkam TMS94 temperature-controlled stage integrated with the Raman instrument. The samples were typically heated at a rate of 20 K/min.

Microscopy characterizations

Optical microscopy was performed using a Nikon SMZ800 microscope. SEM analysis was carried out employing a Quanta FEG 250 instrument, operating at an acceleration voltage of 30 kV within a high vacuum environment (8×10^{-5} mbar). For small cluster SCO core-shell NPs (<1 μm), the sample was deposited as a small (<10 μL) droplet of the aqueous nanocluster suspension onto a glass slide which had been Au-coated through a TEM grid which acted as a mask and created a grid pattern which allow the positions of individual particles to be indexed. After drying, the individual particles could be optically imaged and selected for SERS analysis. The grid then allowed the same particles to be located within the SEM and imaged at higher resolution. All other samples were deposited on aluminum foil. TEM/EDX characterizations were conducted by a TALOS F200X G2: scanning/transmission

electron microscope (S/TEM). The TEM samples were prepared by dropping core-shell NPs solution onto holey carbon film 300 sample grids and drying in air.

Section 2 Fragmentation and orientation on metal surfaces

The fragmentation of ($Fe(neoim)_2$) on Ag surfaces was discussed in the text [6] but ligand dissociation is much more widely known. It has also been observed in [$Fe(bpz)_2$(phen)][7] on Au where the phen ligand dissociates leaving $Fe(bpz)_2$, and [Fe(pypyr($CF_3)_2)_2$(phen)] on Au which gives [Fe(pypyr-($CF_3)_2$)]$^+$ and phen, which is detected on the surface[8].

Changes in spectra with concentration are common for planar organic ligands, which will typically lie flat in low concentration but will adopt a more upright orientation at higher concentrations due to packing requirements, as illustrated in Figure 3A and 3B[9]. The similarity between the Type I spectra of the complex **1** and the low concentration ligand spectra suggests that the Type I spectra arise from free ligand which is created by fragmentation of the complex and lies flat on the surface. Conversely, the Type II spectra of the pressed complex (Figure 3C), resemble the high concentration ligand spectra (Figure 3B, upright orientation) suggesting that the structure of **1** which gives the Type II spectra also has 1,2,4-triazole ligands which are tilted upright, rather than lying flat on the surface. It is interesting to note that this type of behaviour has also been reported for 1,2,4-triazole adsorbed on Cu surfaces, where the ligands are believed to sit upright, coordinating to the metal ion through the nitrogen at 4 position, this structure is believed to be stabilized by hydrogen bonding to neighboring 1,2,4-triazole ligands[10].

Section 3 Estimation of the laser spot size based on Gaussian beam focusing theory

For bulk samples and large clusters the laser was focused onto the sample using a 10× microscope objective with a numerical aperture (NA) of 0.25. The theoretical minimum beam waist radius can be estimated using the diffraction-limited focusing formula:

$$\omega_0 = \frac{\lambda}{\pi \cdot NA}$$

where λ is the laser excitation wavelength (785 nm).

$$\omega_0 = \frac{785nm}{\pi \cdot 0.25} \approx 1.0\ \mu m$$

This gives a focal spot radius of approximately $\omega_0 \approx 1\ \mu m$.

For small clusters a 100× microscope objective with a numerical aperture (NA) of 0.9 giving $\omega_0 \approx 300$ nm was used.

Section 4 Estimation of hot spot volume

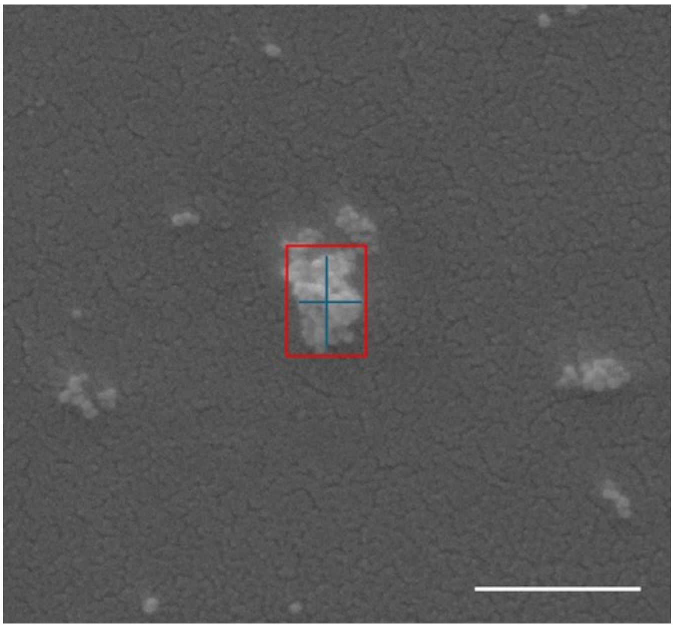

Representative SEM image of an Au@SCO cluster used for hotspot volume estimation. Scale bar represents 500 nm.

Step 1: Estimating the Cluster Volume

Based on SEM measurement, the cluster has a rectangular footprint of approximately 278 × 189 nm. Assuming a cuboid geometry with a height corresponding to 2 layers of Au@SCO nanoparticles (70 nm total height), the cluster volume V_{cluster} is estimated as:

$$V_{cluster} = 278 \times 189 \times 70 = 3.68 \times 10^{6}\, nm^{3}$$

Step 2: Estimating the Number of Au@SCO Nanoparticles

Each core–shell nanoparticle has a diameter of approximately 35 nm (28 nm Au core + 3.6 nm shell × 2), corresponding to a particle volume of:

$$V_{Particle} = \frac{4}{3} \times \pi \times (17.5)^{3} = 2.24 \times 10^{4}\, nm^{3}$$

Considering a packing efficiency of 0.64（Random close packing, the estimated number of particles per cluster is:

$$N = \frac{3.68 \times 10^{6}}{2.24 \times 10^{4}} \times 0.64 \approx 105$$

Step 3: Estimating the Number of Interparticle Gaps

Assuming each particle has ~6 neighboring particles (in 3D close packing), the total number of interparticle gaps (counted without duplication) is:

$$N_{gaps} = \frac{6 \times 105}{2} = 315$$

Step 4: Estimating the Number of Effective SERS Hotspots

Assuming that 100% of the interparticle gaps form sub-2 nm plasmonic junctions capable of supporting strong electromagnetic enhancement:

$$N_{hotspots} = 315 \times 1 \approx 315$$

Step 5: Estimating the Total Probed Volume

Approximating this region as a cube with a side length of 2 nm yields a nominal volume of 8 nm^3 per hotspot. In practice, due to the spatial extent of the enhanced electromagnetic field, the effective probed volume is often taken to be slightly larger. For simplicity and consistency with previous reports, we used 10 nm^3 per hotspot as a representative value in our estimations.

$$V_{hotspots} = 315 \times 10 = 3150\, nm^3$$

Step 6: Calculating the Probed Volume Ratio

Finally, the fraction of the cluster volume that contributes to the SERS signal is:

$$\frac{3150}{3.68 \times 10^6} \approx 0.08\%$$

Only ~0.08% of the total cluster volume is actively contributing to the observed SERS signal.

Supplemental Figures and Table

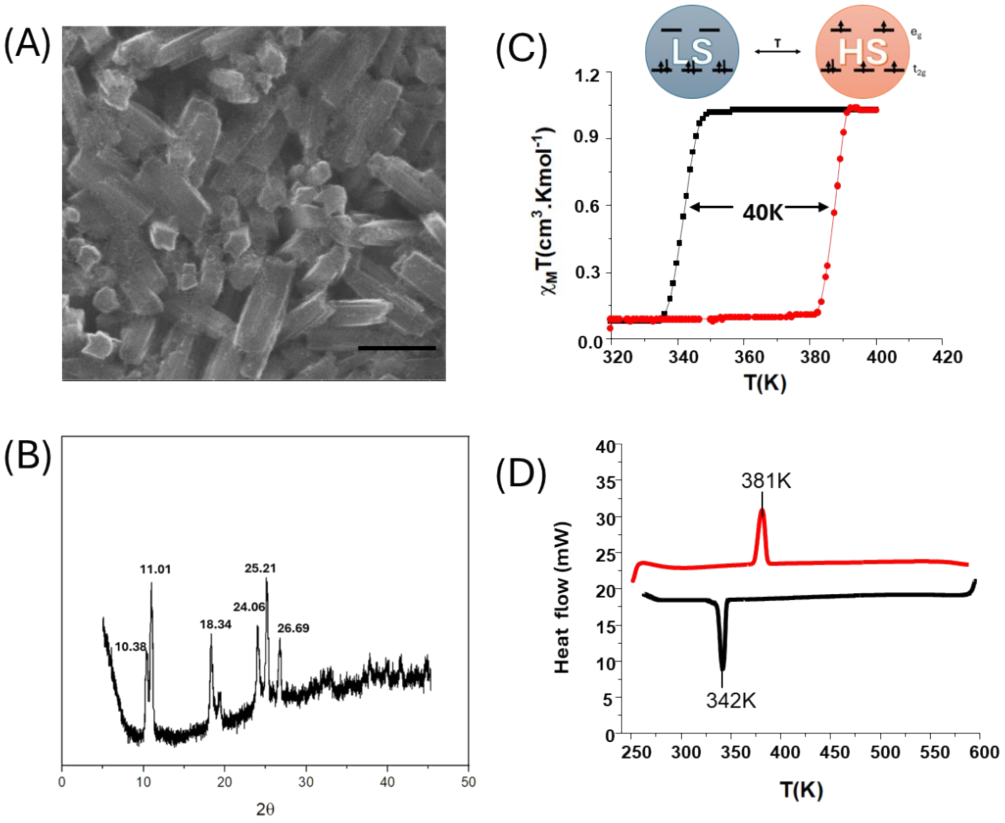

Figure S1. Characterization of $[Fe(Htrz)_2(trz)](BF_4)$

(A) SEM image revealing the rod-like morphology (scale bar 500 nm). (B) Powder X-ray diffraction pattern confirming the presence of the polymorph I structure. (C) Temperature dependence of molar magnetic susceptibility (χ_M), exhibiting a hysteresis loop of 40 K. The upper schematic illustrates the temperature-induced spin-state transition between LS and HS configurations. (D) Differential scanning calorimetry (DSC) curves displaying two distinct thermal peaks.

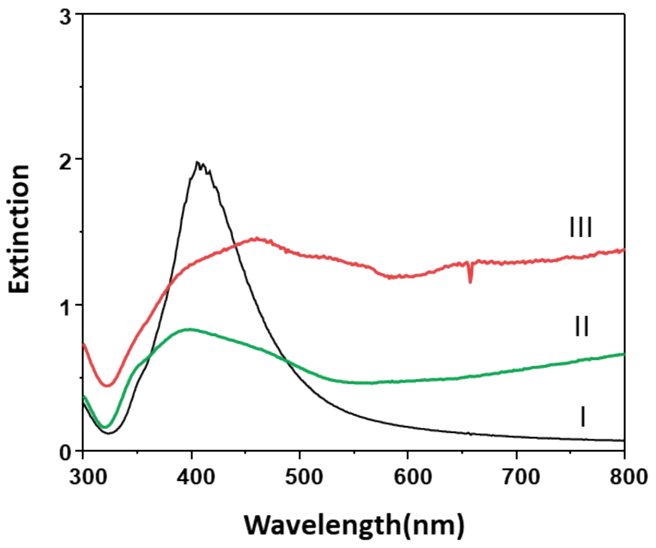

Figure S2. Extinction spectra of Ag nanoparticles and aggregated/array structures

(I), (II) Extinction spectra of Ag colloid before and after aggregation with 1M $(NH_4)_2SO_4$. (III) Extinction spectrum of a nanoparticle array prepared from Ag particles. The spectra in (II) and (III) show strong plasmonic extinction at the 785 nm excitation wavelength used in this work.

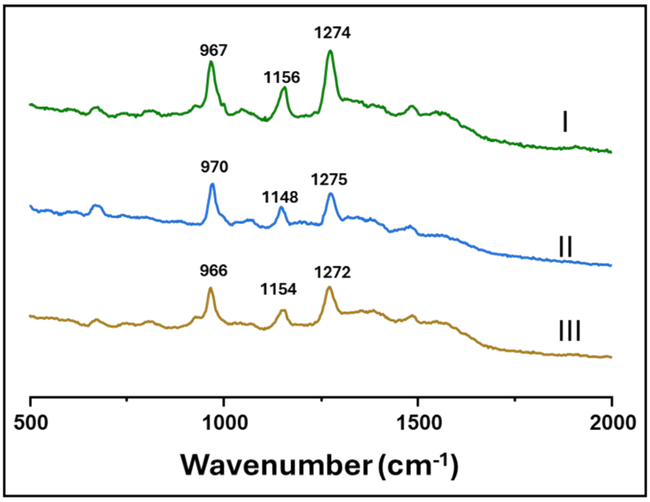

Figure S3. SERS spectra illustrating the similarity of the SERS spectra of triazole ligand obtained from Ag particle enhancing films under 3 different sets of experimental conditions

(I) drop-cast solution of 1,2,4-triazole, (II) pressed 1,2,4-triazole powder, (III) pressed sodium salt of 1,2,4-triazole.

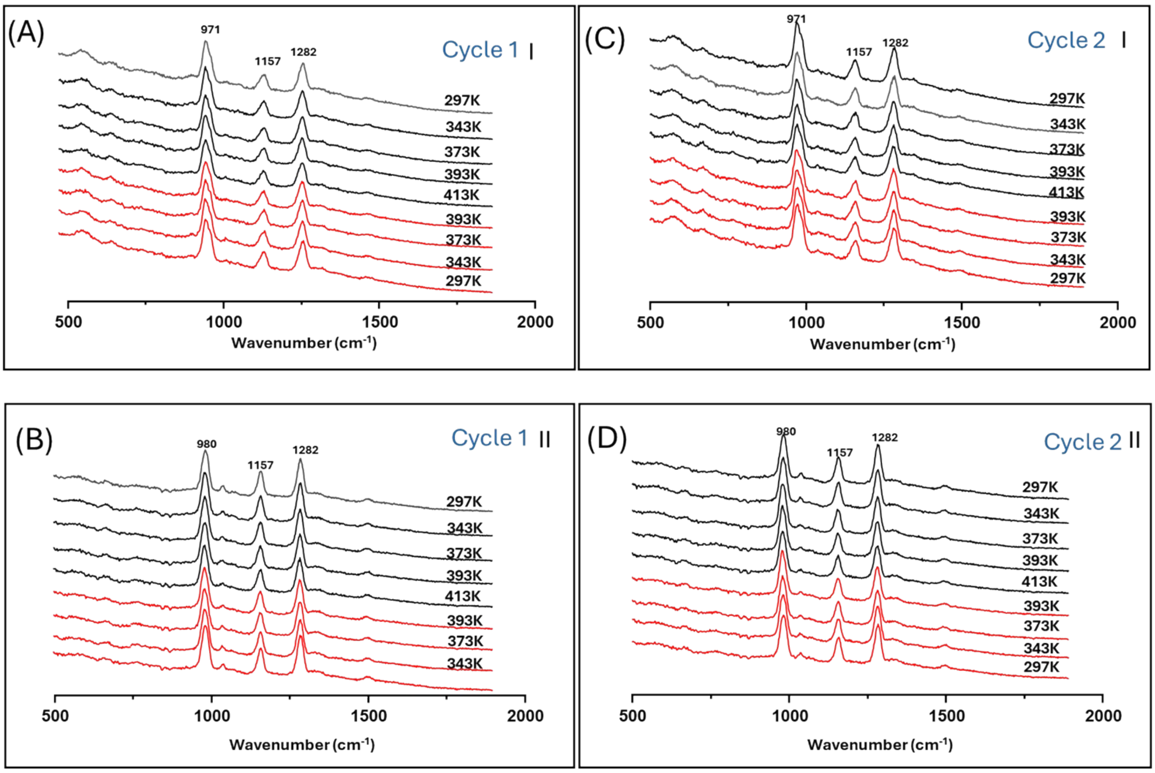

Figure S4. SERS spectra of two types of SCO pressed onto Ag nanosheets under thermal cycling

Type I (A and C) and Type II (B and D) SCO pressed onto an Ag nanosheet, measured over two heating and cooling cycles. No significant peak intensity variations are observed.

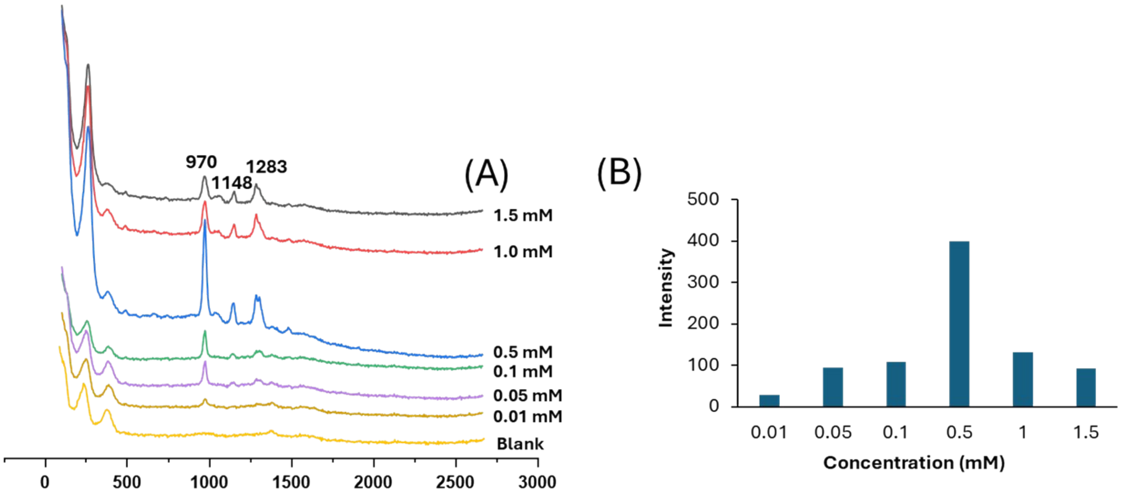

Figure S5. Optimization of triazole ligand concentration for surface functionalization of Au nanoparticles in SCO shell synthesis

(A) SERS spectra of Au nanoparticles used in step 1 of the preparation of core-shell SCO particles treated with the stated concentration of 1,2,4-triazole ligand. (B) Plot of the intensity of the peak at 970 cm^{-1} vs concentration of ligand. The intensities are maximum at 0.5 mM since this is the point where the balance between obtaining the highest surface coverage while not hindering aggregation by sterically protecting the particles is reached.

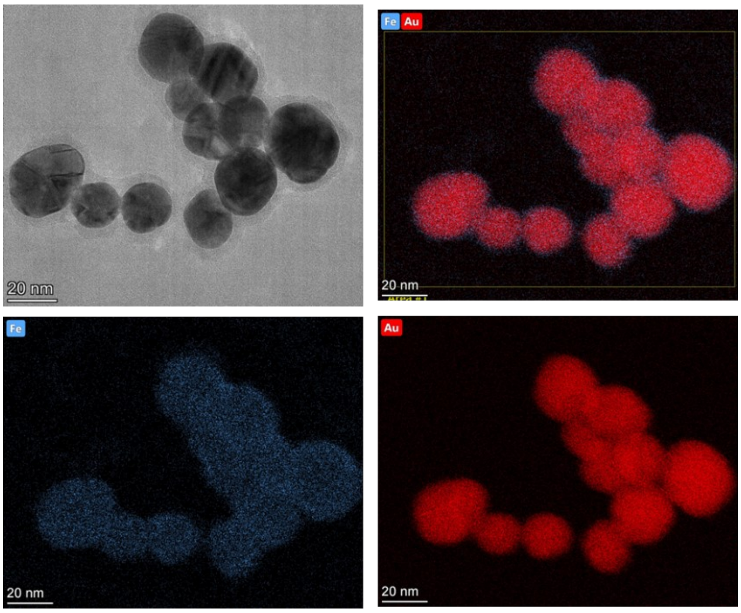

Figure S6. TEM/EDX images of small aggregates of Au@ SCO core-shell nanoparticles

TEM/EDX images showing the Au particles are fully covered with a SCO shell.

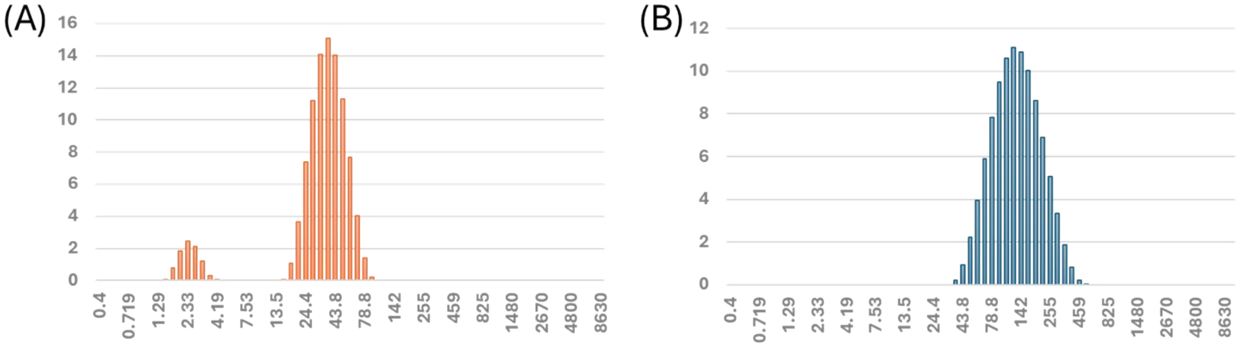

Figure S7. Dynamic light scattering measurements showing the size distribution

(A) Au and (B) Au@SCO nanoparticles, confirming aggregation occurs during shell deposition process.

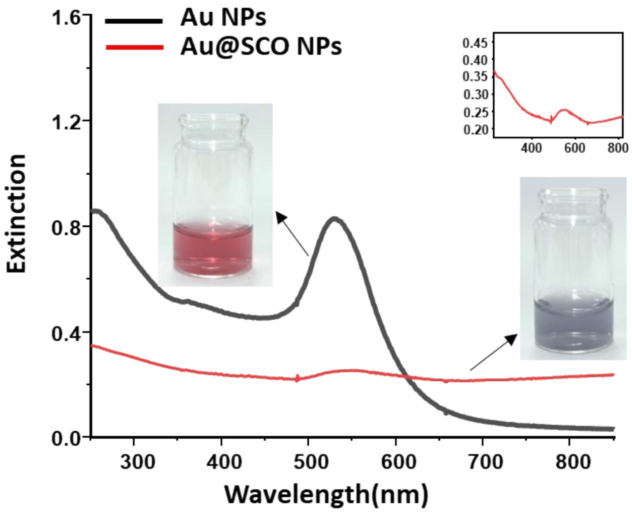

Figure S8. UV-Vis extinction spectra of Au and Au@SCO core-shell nanoparticles.

The insets show optical images of the suspension, illustrating the color change from red to dark purple/grey after shell formation, and an expanded view of the extinction spectrum of the Au@SCO core-shell nanoparticles.

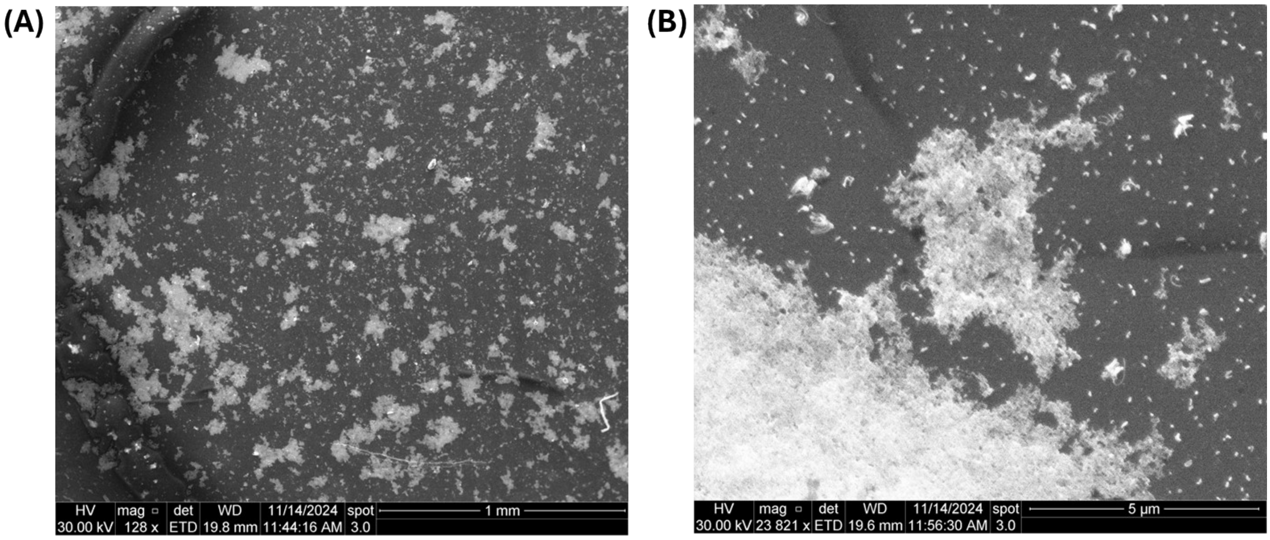

Figure S9. SEM image of "bulk" aggregated Au@SCO nanoparticles

(A) Low-magnification and (B) high-magnification SEM images of aggregated ("bulk") Au@SCO nanoparticles.

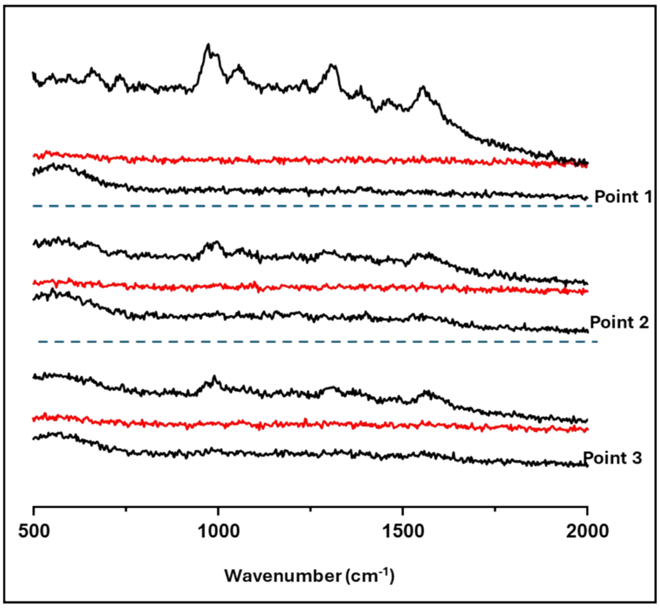

Figure S10. SERS spectra of Au@trizole ligand recorded during a single heating cooling cycle (303 -383 -303 K)

Measurements were recorded at three different sample positions. Signal loss was observed on heating but no recovery of the SCO signal was observed on cooling. This contrasts with the samples Au@SCO where signal loss on heating was followed by recovery on cooling.

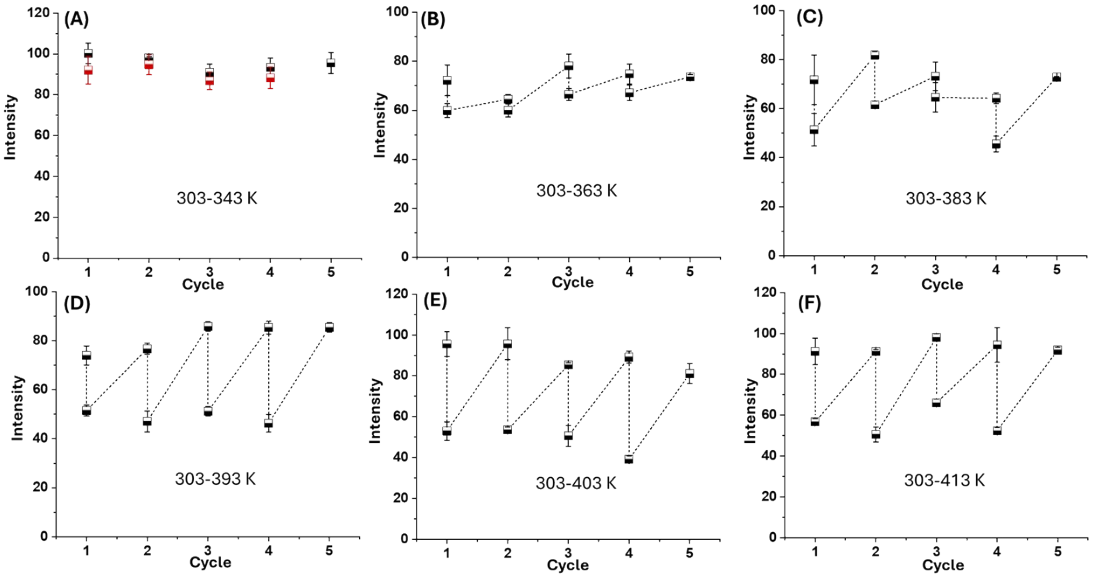

Figure S11. SERS monitoring of thermally induced changes in the 977 cm⁻¹ band during repeated heating cycles

Representative SERS intensity plots showing how intensity of the 977 cm^{-1} band changed on heating from an initial temperature of 303 K to the target temperatures shown on the plots (343 K, 363 K, 393 K, 403 K and 413 K). Each plot shows multiple thermal cycles for a single target temperature cycle allowing the average change to be determined despite the drift in absolute signal over the course of the measurement. Error bars show variation in signal recorded at each temperature during one cycle and are ±1σ.

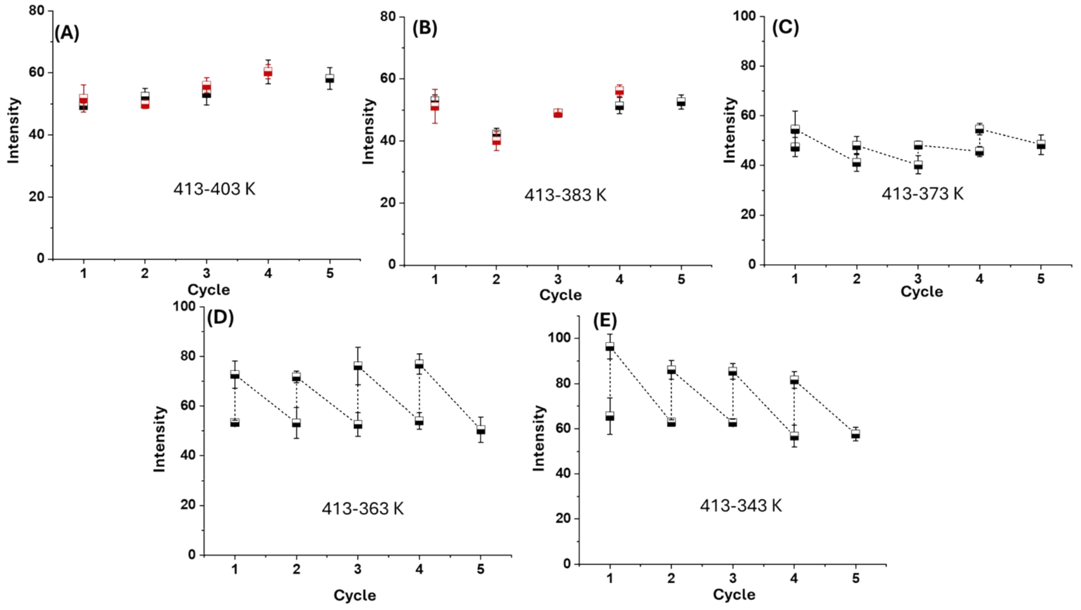

Figure S12. SERS monitoring of thermally induced changes in the 977 cm⁻¹ band during repeated cooling cycles

Representative SERS intensity plots showing how intensity of the 977 cm^{-1} band changed on cooling from an initial temperature of 413 K to the target temperatures shown on the plots (403 K, 383 K, 373

K, 363 K, and 343K). Each plot shows multiple thermal cycles for a single target temperature cycle allowing the average change to be determined despite the drift in absolute signal over the course of the measurement. Error bars show variation in signal recorded at each temperature during one cycle and are ±1σ.

The conversion at each temperature point was calculated using the equation $|(I_{\text{Initial}}-I_{\text{T}})|/I_{\text{Initial}}$. Furthermore, the thermal hysteresis behaviour was quantified by analyzing the temperature-dependent HS conversion using the Boltzmann fitting function in Origin software, expressed as follows:

$$y = A_2 + \frac{A_1 - A_2}{1 + e^{\frac{x - x_0}{dx}}}$$

where y represents the normalized HS conversion, $A_1$and A_2 correspond to the asymptotic lower and upper limits, x_0 is the inflection point (temperature at 50% conversion), and dx is the slope factor describing the transition sharpness.

Table S1 Fitting results from Boltzmann equation

Parameters	Heating	Cooling
A_1	-0.006 ± 0.065	0.008 ± 0.024
A_2	0.943 ± 0.136	0.987 ± 0.019
x_0	107.02 ± 5.91	99.26 ± 0.36
d_x	8.64 ± 4.65	2.39 ± 0.91
R-Square (COD)	0.980	0.999

The 50% HS conversion temperatures were determined as 381K for heating ($x_{heating}$) and 372 K for cooling ($x_{cooling}$). The thermal hysteresis width (ΔT) was calculated as their difference:

$$\Delta T = x_{heating} - x_{cooling} = 9 \text{ K}$$

Reference

1. M. Palluel, N. M. Tran, N. Daro, S. Buffière, S. Mornet, E. Freysz and G. Chastanet, *Adv. Funct. Mater.*, 2020, **30**, 2000447.
2. Y. Xu, M. P. Konrad, W. W. Lee, Z. Ye and S. E. Bell, *Nano Lett.*, 2016, **16**, 5255-5260.
3. G. Frens, *Nature, Phys. Sci.*, 1973, **241**, 20-22.
4. P. Lee and D. Meisel, *J. Phys. Chem.*, 1982, **86**, 3391-3395.

5. R. Torres-Cavanillas, R. Sanchis-Gual, J. Dugay, M. Coronado-Puchau, M. Gimenez-Marques and E. Coronado, *Adv. Mater.*, 2019, **31**, e1900039.
6. S. Johannsen, M. Gruber, C. Barreteau, M. Seredyuk, J. Antonio Real, T. Markussen and R. Berndt, *J. Phys. Chem. Lett.*, 2023, **14**, 7814-7823.
7. T. G. Gopakumar, M. Bernien, H. Naggert, F. Matino, C. F. Hermanns, A. Bannwarth, S. Muhlenberend, A. Kruger, D. Kruger, F. Nickel, W. Walter, R. Berndt, W. Kuch and F. Tuczek, *Chemistry*, 2013, **19**, 15702-15709.
8. S. Rohlf, J. Grunwald, T. Jasper-Toennies, S. Johannsen, F. Diekmann, M. Studniarek, R. Berndt, F. Tuczek, K. Rossnagel and M. Gruber, *J. Phys. Chem. C*, 2019, **123**, 17774-17780.
9. E. Papadopoulou and S. E. J. Bell, *J. Phys. Chem. C*, 2011, **115**, 14228-14235.
10. M. Muniz-Miranda, F. Muniz-Miranda and S. Caporali, *Beilstein J. Nanotechnol.*, 2014, **5**, 2489-2497.
